# Bridging to systemic therapy: PSMA-PET-based early and repetitive stereotactic body radiotherapy for nodal and bone oligo-recurrent prostate cancer

**DOI:** 10.3389/fruro.2026.1738508

**Published:** 2026-09-07

**Authors:** Arne Grün, Alexandra Partyka, Markus Galler, Torsten Schlomm, Dirk Böhmer, Kurt Miller, Daniel Zips, Goda Kalinauskaite

**Affiliations:** 1 Department of Radiation Oncology, Charité – Universitätsmedizin Berlin, corporate member of Freie Universität Berlin, Humboldt-Universität zu Berlin, and Berlin Institute of Health, Berlin, Germany; 2 Department of Nuclear Medicine, Charité – Universitätsmedizin Berlin, corporate member of Freie Universität Berlin, Humboldt-Universität zu Berlin, and Berlin Institute of Health, Berlin, Germany; 3 Department of Urology, Charité – Universitätsmedizin Berlin, corporate member of Freie Universität Berlin, Humboldt-Universität zu Berlin, and Berlin Institute of Health, Berlin, Germany

**Keywords:** oligo-metastases, prostate cancer, PSMA PET/CT, recurrence, SBRT (stereotactic body radiation therapy)

## Abstract

**Introduction:**

To provide a detailed analysis regarding survival, local control and toxicity of nodal/bone oligo-recurrent prostate cancer identified and treated with PSMA-PET/CT and robotic SBRT using a uniform dose/fractionation scheme. The use of ADT was excluded.

**Methods:**

Retrospective analysis of patients with initial high-risk factors presenting with nodal/bone oligo-recurrent PCA in Ga68- or F18-PSMA-PET/CT treated with 1-3 fraction robotic SBRT.

**Results:**

60 patients/172 metastases were treated in 95 treatment series with 43 patients receiving >1 SBRT course. Median FU was 22 months. bPFS/SBRT-FS/ADT-FS was 6/23/32 months at 89.7% local control and 6.5/1.1% acute/late grade 1 AE. High PSA-nadir, initial pN1-status and longer time to PSA-nadir were predictive for worse bPFS/SBRT-FS.

**Discussion:**

Metastasis-Directed-Therapy using ablative focal SBRT is effective in bridging the gap between the identification of early metastases and the initiation of palliative systemic therapy. It is a fast outpatient procedure yielding high rates of local control, free of significant toxicity and repeatable if necessary. ADT could be withheld for almost 3 years. Patients with predictors for worse outcome could be identified.

## Introduction

In a current single-center analysis of 43 patients with newly diagnosed oligo-metastatic PCA and a follow-up (FU) of up to 10 years, overall survival (OS) was 100% (1). The rationale for metastases-directed therapy (MDT) in this early stage is three-pronged. Genetic analyses showed that the tumor cell clones responsible for fatal dissemination do not necessarily reside within the primary tumor (2, 3) but can emerge from every single metastasis (4). Secondly, early PCA metastases diagnosed with molecular imaging harbor less high-risk DNA features with better survival prognosis (5), and lastly, systemic therapy is effective but toxic [combination arms in STAMPEDE and LATITUDE: 47% and 63% ≥grade 3 adverse events (AEs)] (6, 7) and should thus not be squandered in this early stage of dissemination (8). Current PSMA-based imaging detects oligo-metastases at very low PSA counts (9). MDT improves progression-free survival (PFS) and androgen deprivation therapy-free survival (ADT-FS) (10), but the data are tainted by the use of outdated diagnostics and mixed-approach MDT [surgery, conventional external beam radiotherapy (RT), inhomogeneous dose concepts (11), and the use of ADT (12–17)].

We analyzed a large cohort with nodal/bone OR disease with regard to survival, toxicity, and local control. Patients were identified/treated with modern tracer positron emission tomography/computed tomography (PET/CT) and robotic stereotactic body radiotherapy (SBRT) using a defined dose concept. Patients on ADT were excluded. We hypothesize that early/potentially repetitive SBRT yields substantial freedom from ADT at low AE rates even in patients with initial high-risk features. We provide a detailed analysis of prognostic factors with regard to survival endpoints.

## Methods

**Figure 1:**
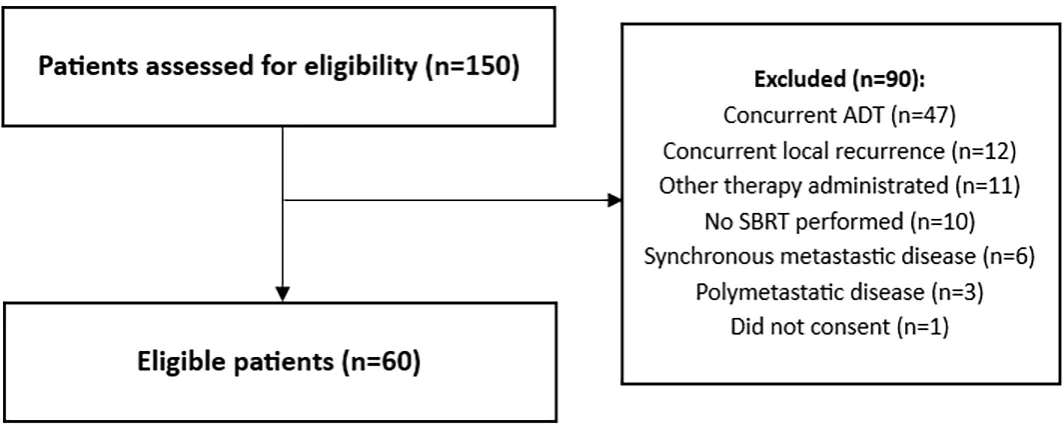
Patient enrollment. We screened 150 consecutive patients regarding our inclusion/ exclusion criteria. We included patients with modern tracer-PSMA-PET/CT-based diagnosis of 1-5 oligo-recurrent metastases and a PSA count of up to 5 ng/ml. No additional therapy was allowed. Patients with concomitant local recurrences and patients receiving non-robotic SBRT were excluded.

Retrospective analysis of patients treated with robotic SBRT [CyberKnife (CK), Accuray Inc., Sunnyvale, CA, USA] for nodal/bone OR PCA at the Charité – University Medicine Berlin Department of Radiation Oncology and Radiotherapy (**Figure 1**). All treatments were conducted according to Good Clinical Practice and the German Radiation Protection Laws. The research complied with the Declaration of Helsinki. The Charité ethics board approved this study (XXX). Since data were stored anonymously, the informed consent requirement was waived.

An unspecified PSA recurrence after either surgery (RPE) ± RT or definitive RT had triggered PSMA-PET imaging with the subsequent identification of nodal/bone OR. The metastasis constituted the GTV with a 5-mm margin for PTV. The system uses two stereoscopic x-rays for either bone or fiducial marker tracking. Technique and constraints were according to published data (18–23). AEs were scored using Common Terminology Criteria of Adverse Events Version 4 (CTCAEv4). Endpoints were bPFS, SBRT-FS, ADT-FS, toxicity, and local control (if applicable). bPFS was defined as PSA increase from nadir of >0.2 ng/mL or death; SBRT-FS was defined as the time between the first and next course of SBRT (if applicable) or death. Repat SBRT was offered in case of further oligo-metastatic recurrence. There was no predefined upper limit; repeat SBRT cycles usually came to an end with the initiation of ADT. There, ADT-FS was defined as the time between the first course of SBRT and the initiation of ADT or death. PSA controls were trimonthly during FU; all patients were scanned due to an increase in PSA but there was no predefined PSA value triggering re-staging/re-SBRT/initiation of ADT; hence, the data reflect the treating physicians’ discretions. No patient had symptomatic disease.

The data analysis was conducted using SPSS V29 (IBM, Armonk, NY, USA). The report follows the Strengthening the Reporting of Observational Studies in Epidemiology (STROBE) statement (24). The Kaplan–Meier estimator was used to analyze time-to-event endpoints. Cox regression was used to evaluate prognostic factors on ADT-FS, bPFS, and SBRT-FS. Covariates deemed significant in univariate analysis were entered into multivariable Cox regression analysis. Repeat treatments were handled as individual cases. *p*-values <0.05 were considered to be significant.

## Results

**Figure 2:**
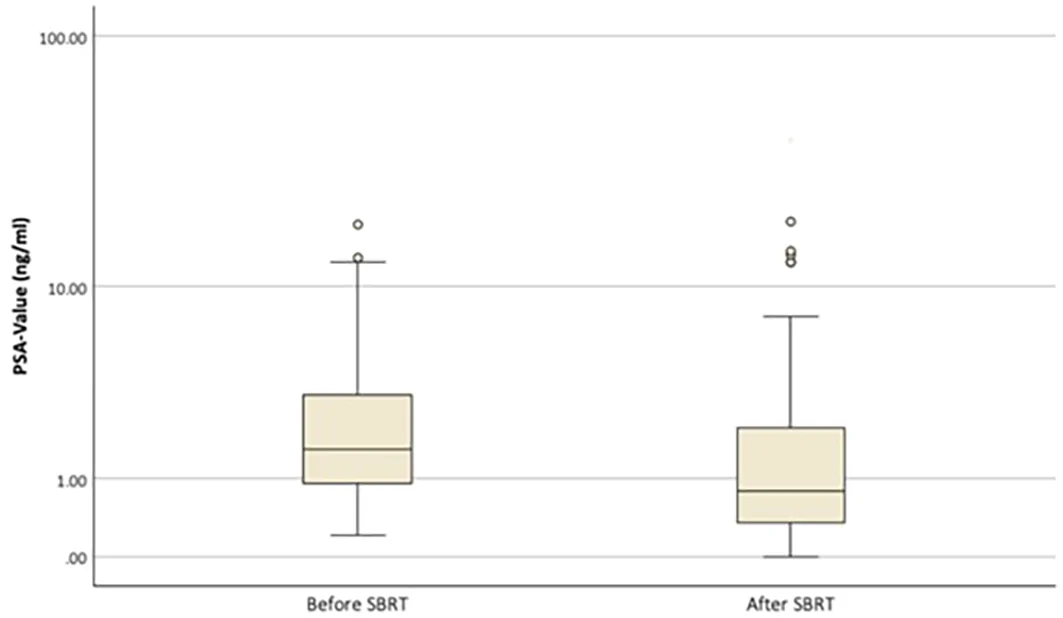
PSA-response. The median pre-SBRT-PSA was 1.66 ng/ml (0.95- 3.21ng/ml). A vs. no PSA-response was detected in 67 (70.5%) vs. 23 (24.2%) series. The median nadir was 0.58 ng/ml (0.21-1.27ng/ml). Median nadir after the 1st/2nd series was 0.5/0.58 ng/ml. Median time to nadir was 4.27 months. (for better legibility the y-axis is scaled logarithmically).

**Figure 3:**
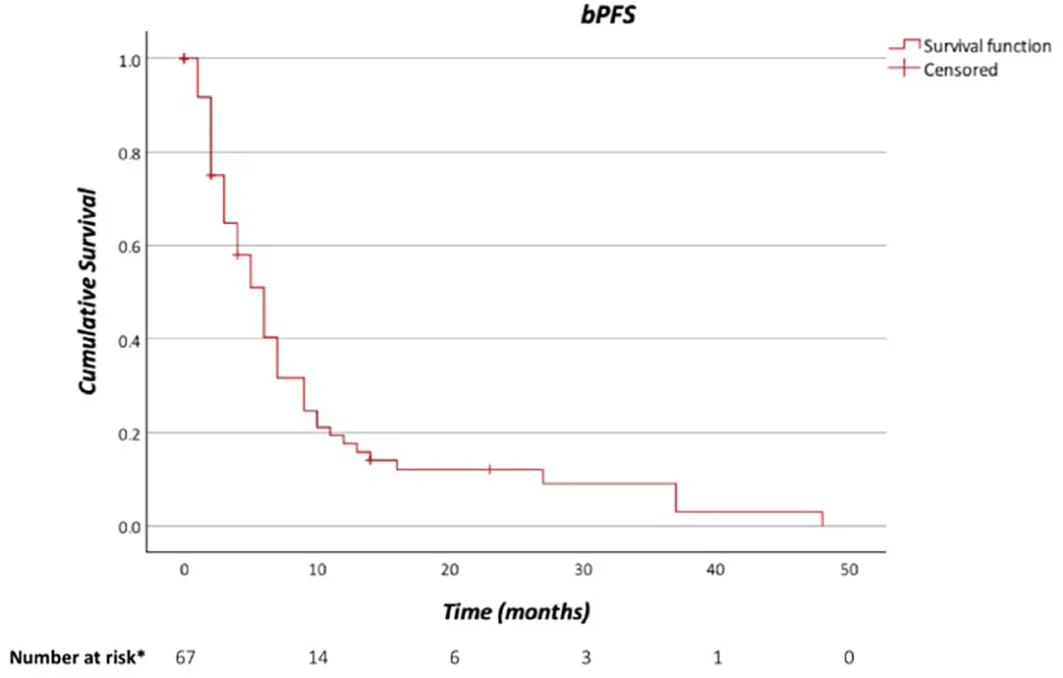
Biochemical (PSA) Progression-Free-Survival (bPFS). Median overall bPFS was 6 (4.56-7.44) months (this graph). In nodal-/ bone-only and mixed cases bPFS was 6 (4.73-7.27)/ 2 (0.08-5.92) and 4 months (1.85-6.15). Six- and 12-mo bPFS was 40.0% and 17.6%. bPFS after the 1st and 2nd SBRT series was 6 (4.79-7.21) and 6 (1.43-8.57) months.

**Figure 4:**
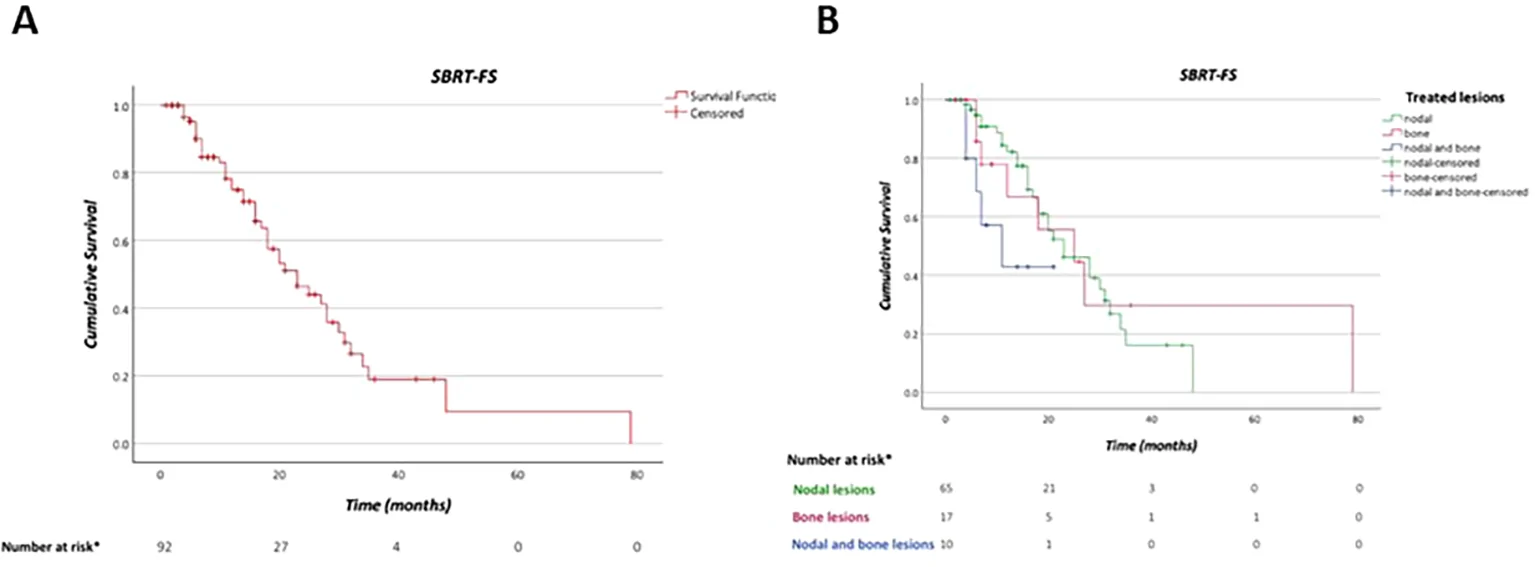
Stereotactic-Body-Radiotherapy-Free Survival (SBRT-FS). Median overall **(A)**, nodal, bone and mixed **(B)** SBRT-FS was 23 (16.00-30.01), 23 (15.06-30.94), 25 (5.36-44.65) and 11 (1.42-20.57) months with 43 patients receiving repeat SBRT. Six, 12 and 24 months SBRT-FS was 90.1%, 75.0% and 46.5%. Median SBRT- FS after the 1st and 2nd series was 23.0 (15.49-50.51) and 28 (9.99-46.01) months.

**Figure 5:**
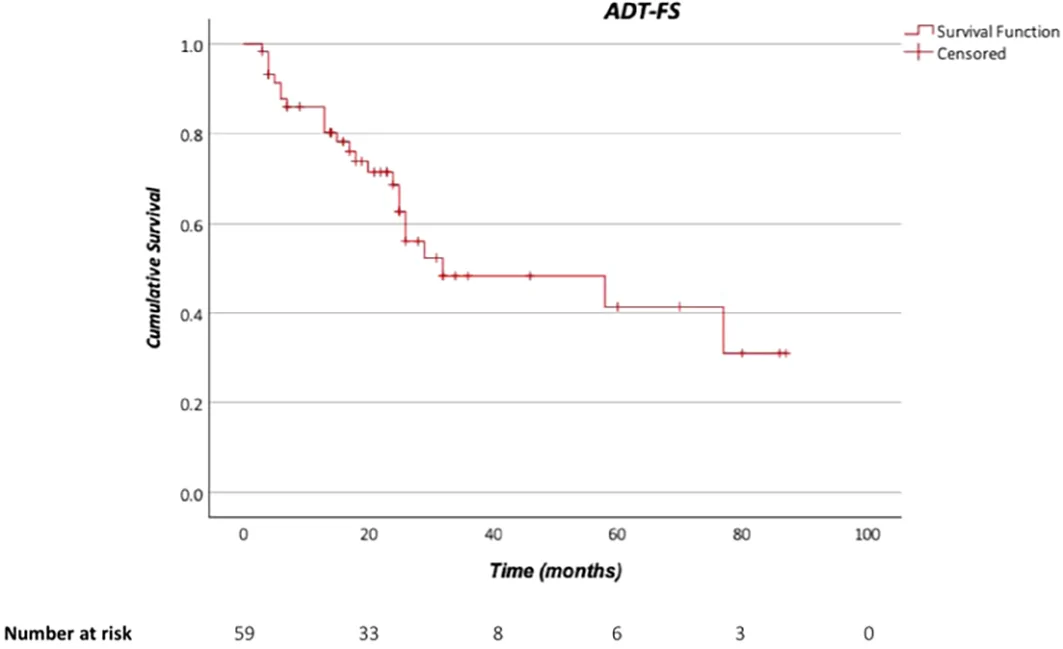
Androgen-Deprivation-Therapy-Free-Survival (ADT-FS). Twenty patients (33.3%) needed no further treatment. ADT was initiated in 23 patients (38.3%), 5 (8.3%) received further non-robotic SBRT, 3 had polymetastatic progression without a decision on further treatment, 1 had surgery and 1 hyperthermia. Seven patients were lost to FU. Median ADT-FS was 32 (0.00-68.41) months. ADT-FS at 1- and 2-years was 86.0% and 68.5%.

For patients’ characteristics at initial diagnosis and treatment, see **Table 1**. Between November 2013 and December 2021, 60 patients/172 metastases were treated in 95 series. For patients’ characteristics at the time of SBRT, see **Tables 2**, **3**. Bone tracking was used in the majority of cases (92.4%). Marker tracking was used in seven cases (7.6%) due to pelvic lymph nodes not adhering to pelvic wall. Median FU was 22 (13.0–31.0) months. Twenty patients (33.3%) reached the end of FU without any further treatment. Median overall post-SBRT nadir was 0.58 ng/mL. Median time to nadir was 4.27 months (**Figure 2**, **Table 4**). Toxicity was mild (**Table 5**). Local control in patients with repeat PET/CT was 89.7% in 117 metastases (nodal: 93.5%; bone 76%). Median bPFS was 6 (4.71–7.29) months (**Figure 3**, **Supplementary Tables 6.1**, **6.2**). Median SBRT-FS was 23 (15.47–30.53) months. Forty-three patients received repeat SBRT (**Figure 4**; **Supplementary Tables 7.1**, **7.2**). Median ADT-FS was 32 (0.00–68.43) months (**Figure 5**; **Supplementary Table 8**). ADT-FS at 1 and 2 years was 85.9% and 68.4%, respectively.

**Table 1:** Patients' characteristics at initial diagnosis and treatment (n=60).

Age (years)	median (IQR) 61.0 (56.0-69.0)
Initial PSA (ng/ml)	median (IQR) (n=56) 11.39 (6.98-17.67)
Biopsy Gleason-score	n (%)
6	3 (5)
7	1 (1.7)
7a	7 (11.7)
7b	8 (13.3)
8	15 (25)
9	10 (16.7)
10	1 (1.7)
n/a	15 (25)
1st-line therapy	n (%)
RPE	55 (91.7)
* adjuvant radiotherapy*	32 (58.2)
* no radiotherapy*	22 (40.0)
* n/a*	1(1.8)
Radiotherapy	4(6.7)
* Salvage RPE*	3(75)
* no RPE*	1 (25)
* HiFu & RPE*	1 (1.7)
Post-op Gleason-score (n=50)	n(%)
6	1 (1.7)
7a	9 (15.3)
7b	16 (27.1)
8	10 (16.9)
9	16 (27.1)
10	1 (1.7)
n/a	6 (10.2)
pT-stage (n=59)	n (%)
pT2a	1(1.7)
pT2b	2(3.4)
pT2c	15 (25.4)
pT3a	11 (18.6)
pT3b	25 (42.4)
ypT3b	1(1.7)
n/a	4(6.8)
pN-stage (n=59)	n (%)
pN0	42 (71.2)
pN1	12 (20.3)
pos tive nodes	0.11 (0.07-0.2)
n/a	5 (8.5)
Resection status (n=59)	n (%)
RO	33 (55.9)
R1	21 (35.6)
R2	1(1.7)
n/a	4(6.8)
Prior ADT	n (%)
yes	13 (21.7)
no	47 (78.3)

Predominant and second most biopsy and post-op Gleason-scores were 8 (25%), 9 (16.7%) and 7b, 9 (each 27.1%). 42.4% of patients had a post-surgery T-stage of pT3b, 20.3% had positive nodes and 35.6% an incomplete resection. 21.7% were on ADT at least 6 months prior to SBRT. A high proportion of patients were at high risk for recurrence after first-line therapy.

**Table 2:** Patients' characteristics at SBRT (n=95 series).

Age at first SBRT (years)	Median (IQR) 69 (61.25-75.0)
Time between first diagnosis of PCA and first SBRT	Median (IQR) 60.9 (35.07-95.68)
PSMA-PET-CT-Ligand	n (%)
^65^Ga	73 (76.8)
^18^F	21 (22.1)
*n/a*	1 (1.1)
Metastases	n (%)
Nodal	138 (80.2)
Bone	34 (19.8)
Total	172 (100.0)
Type of metastases per series	n (%)
Nodal	68 (71.6)
Bone	17 (17.9)
Total	10 (10.5)
Metastases per series per patient	n (%)
1	47 (49.5)
2	32 (33.7)
3	10 (10.5)
4	2(21)
5	2(21)
6	1(1.1)
7	1 (1.1)
Median	2.0
Localization of metastases (n=166)	n (%)
Lymph-nodes	138 (80.2)
Pelvic	112(81.2)
Abdominal (para-aortal)	26(18.8)
Bone	34(19.8)
*Pelvic incl. sacral spine*	15 (44.1)
*Lumbar spine*	4(11.8)
*Thoracic spine/ribs*	14(41.2)
*Scapula*	1 (2.9)
PET/CT to SBRT time (months)	Media (IQR) 2.0 (1.4 -2.93
Pre-SBRT PSA (ng/ml)	Median (IQR) 1.66 (0.95-3.21)
PSA-response	n(%)
Yes	67 (70.5)
No	23 (24.2)
n/a	5(5.2))
Post SBRT PSA-nadir (ng/ml) (n=66)	Median (IQR) 0.58 (0.21-1.27)
PSA-nadir after 1^st^ SB RT series (ng/ml) (n=41)	Median (IQR) 0.5 (0.09-0.88)
PSA-nadir after 2^nd^ SBRT series (if applicable) (ng/ml) (n=23)	Median (IQR) 0.58 (0.13-0.92)
Time to PSA-nadir (months) (n=66)	Median (IQR) 4.27 (2.58-6.44)

Two patients had 1 and 2 additional suspicious lymph-nodes compared to the PET/CT, hence 1 patient had 6 and 1 patient 7 metastases. Median number of metastases per series was 1.5. Mostly, 1 (50%) or 2 metastases (33.7%) were treated. Patients with ≤5 metastases detected using modern-tracer PET/CT and a PSA ≤5ng/ ml were included.

**Table 3:** Volumes and doses.

	All (n=172)	Lymph-node metastases (n=137)	Bone metastases (n=35)
PTV volume (cm^3^). Median (IQR)	3.31 (1.88-6.62) in n=171*	2.89 (1.73-4.86)	6.95 (3.39-12.66) in n=34*
GTV volume (cm^3^). Median (IQR)	0.77 (0.31-1.89)	0.58 (0.23-1.36)	2.29 (0.92-3.75)
Minimum dose (Gy). Median (IQR)	21.85 (19.69-23.0)	22.34 (20.69-23.09)	19.35 (18.7-20.75)
Median dose (Gy). Median (IQR)	27.85 (24.97-28.7)	28.17 (25.52-28.74)	25.18 (24.01-25.93)
Maximum dose (Gy). Median (IQR)	33.99 (29.66-34.29)	34.14 (30.0-34.29)	30.0 (28.57-30.0)

Dose to the PTV-surrounding 70% isodose line was either 1 x 18-21 Gy or 3 x 8 Gy. Spine to fiducial tracking ratio was n=85/7. *missing data on one metastasis. The metastases constituted the gross target volume (GTV) with a 1-5 mm isometrical planning target volume PTV (location-dependent).

**Table 4:** Adverse events: Maximum post-SBRT AE were grade 1 (fatigue: n=5; diarrhea: n=3; nausea: n=2; pain, proctitis, polyuria: n=1).

Acute adverse events	n (%)	Late adverse events	n (%)
None	86 (93.5)	none	86 (93.5)
Grade 1	6 (6.5)	Grade 1	1 (1.1)
		missing	5 (5.4)

One patient had late symptoms with a persistently painful bone metastasis.

**Table 5:** PSA response in cases with PSA response (A) and with in cases without PSA response i.e.

A: Cases with PSA-response			B. Cases with PSA-progression		
	PSA-pre-SBRT (ng/ml) Median (IQR)	PSA-Nadir (ng/ml) Median (IQR)		PSA pre-SBRT (ng/ml) Median (IQR)	PSA post-SBRT* (ng/ml) Median (IQR)
Type of treated metastases			Type of treated metastases		
node (n=49)			node (n=14)		
bone (n=12)	1.55 (1.07-4.64)	0.63 (0.29-1.32)	bone (n=4)	1.41 (0.58-3.65)	2.42 (1.73-5.78)
both (n=5)	1.56 (0.83-3.06)	0.30 (0.08-0.62)	both (n=5)	251(1.08-14.30)	8.32 (2.27-32.9)
	2 15 (1.17-2.84)	1.54 (0.16-2.54)		1.05 (0.85-4.56)	4.42 (1.53-12.97)
Number of metastases			Number of metastases		
1 (n=36)	1.10 (0.76-3.06)	0.39 (0.10-0.77)	1 (n=8)	210 86-11.72)	3.82 (2.10-13.28)
2 (n=20)	2.23 (1.60-4.39)	1.37 (0.45-2.60)	2 (n=10)	0.97 (0.58-2.76)	2.16 (1.52-9.08)
3 (n=6)	1.55 (1.40-2.17)	0.71 (0.44-1.40)	3 (n=3)	1.45	4.42
4 (n= 1)	1.05	0.78	4 (n=1)	5.63	5.7
5 (n= 1)	5.09	0.11	5 (n=1)	26	287
6 (n= 1)	11,2	0.45			
7 (n= 1)	0.46	0.08			

PSA-progression (B) stratified by type and number of metastases.

## Discussion

Early modern-tracer-PET-based focal SBRT is an efficient outpatient procedure absent of significant toxicity. We treated 60 patients/172 metastases. Werensteijn-Honigh et al. treated a larger cohort but a lower number of metastases (25). Ours is the second largest cohort of metastases treated after Bowden et al. (199 patients) (26). Our median FU was 22 months, which is similar to other analyses on the subject (27, 28). Patients received Ga68- (29) or F18- (30) PSMA-PET/CT. Older PET tracers (choline) are inferior to PSMA (especially at low PSA counts) (31–36), although in the OSPREY trial, the primary endpoint for sensitivity was not met (37). Ga^68^- vs. ^18^F-choline-based SBRT is superior with respect to ADT-FS, although the median PSA in both groups was slightly thwarted in favor of Ga^68^ (38). We initiated SBRT at a median PSA of 1.64 ng/mL. Other groups were treated at higher PSA values [STOMP, MDT-arm: 5.3 ng/mL (12); Siva et al. 6.4 ng/mL (13); ORIOLE-trial: up to 50 ng/mL (15)], which is in contrast to Deek et al. suggesting low-volume disease (low number/low-volume GTV) as the optimal time for treatment (39). The PSA response rate in our cohort was 71.4%. Studies with higher response rates combined MDT and ADT (13, 26, 39, 40). We excluded patients on ADT within the last 6 months prior to SBRT to minimize residual ADT effects. Six months was a pragmatic compromise although testosterone recovery might be faster/slower depending on the individual patient and duration of ADT (41). We used 1–3 fraction robotic SBRT. Others included elective volume treatment (pelvic LNE/elective RT) (12, 15, 17, 42, 43). As a consequence, Vaugier et al. reported 13.4%/14.9% grade 2 acute GU/GI and 4.4% grade 3 acute GU AE (44).

Our bPFS was 6 months (4.71–7.29) compared to Ost et al. with 10 months but with a less strict definition of PSA recurrence (PSA increase >25% or >2 ng/mL) (12). Kneebone et al. had a similar definition, but a bPFS of 11 months in a cohort comprising only 33% patients with aggressive histology compared to 63% in our cohort (14). In the OLIGOPELVIS trial, PFS was 25.9 months at a higher rate of AE/longer treatment time/exclusion cN1 (16). Corroborating Cozzi et al., we showed that a higher PSA nadir was associated with shorter bPFS (45). SBRT-FS after the first/second series was 6 months compared to 16 and 8 months in Henkenberens et al., but they treated nodal and bone metastases with high-volume conventional RT, resulting in increased acute and late AE (46). Time to re-SBRT data are sparse. Deek et al.’s time to next intervention (including a second SBRT course) was 14 months (39). We demonstrate a 23-month SBRT-FS in patients with multiple series (23/28 months after the first/second series, respectively). Initial pN1-status was negatively prognostic while longer time-to-nadir was positively prognostic for SBRT-FS.

Our median ADT-FS was 32 (0–68.43) months. One-third (33.3%) of our patients reached the end of FU without therapy escalation. In the STOMP trial, ADT-FS was 21 months (12). Siva et al. described a subgroup with an ADT-FS rate at 2 years of 48% (13) compared to 68.4% in our study. In Glicksmann et al., median time to ADT was not reached at a median FU of 15.9 months (17). Mazzola et al. reported on an ADT-FS of 13.5 months in a cohort of bone-only oligo-metastases (47).

Metastasis-free survival in the PEACE V STORM trial (SBRT + stADT vs. ENRT + stADT) was 63% vs. 76% at a low rate of grade 3 AEs (48). A total of 117 metastases could be analyzed with respect to local control. PET-assessed local control was 89.7%/93.5%/76% for the whole cohort/nodal/bone metastases. Data on local control varies between 86.6% and 93% (13, 39, 40, 49). Inferior local control in bone vs. lymph node metastases could be attributable to the protective microenvironment of the bone marrow for cancer cells and/or hypoxia. Aslot et al. have shown similar results (50).

We saw no AE grade >1. 6.5% and 1.1% of patients had acute and late grade 1 AE. Werensteijn-Honingh et al. and Siva et al. had 44% and 67% acute (grade 1–3) AE, grade 3 being a vertebral body fracture (13, 25). Temporary fatigue was the most frequent AE in our cohort and the literature (13, 25). New concepts like temporary intensified hormone therapy maintained low PSA counts even after testosterone recovery but were still plagued with high rates of grade 2 and 3 hormone therapy-related AE (51). The RADIOSA trial showed a doubling of clinical PFS (15.1% vs. 32.2%) with the addition of 6 months ADT to SBRT with mostly grade 1 AEs (52). Early reports on the PERSIAN trial showed a non-statistical trend in favor of the experimental arm with AE data not yet published (53). While the addition of modern anti-hormonal agents (arbiraterone, enzalutamide, apalutamdide, and darolutamide) to standard ADT showed promising survival benefits in multiple subgroups and has not necessarily increased ADT toxicity, reported grade 3–4 AE rates were still in the range of 25%–44% (54–57).

## Limitations

There are critical shortcomings concerning this analysis. The retrospective nature of this work is susceptible to fault and there is a lack of a control arm. Handling repeated SBRT courses as new cases in the Cox regression model might have biased results. Also, the question if early oligo-recurrences will inevitably develop into life-threatening disease is still unanswered (58). Ambiguity regarding the validity of traditional definitions of PSA recurrence after both surgery and RT is reflected in the fact that there was no predefined PSA cutoff value triggering imaging and therapy as well as re-imaging and re-treatment. While reflecting real-life decision-making by the treating physicians, the data on SBRT-FS and ADT-FS might be skewed by the individual treating physician’s discretion. Current recommendations propose a risk-adapted approach in defining clinically relevant recurrences incorporating not only the absolute PSA value but also high-risk features such as initial grade group, time to recurrence, and dynamics of PSA increase (59, 60). Taking all of this into account, one might argue that for some of our patients presenting with gradual disease progression, observation would have also been an option. Ultimately, it remains to be seen if SBRT-FS and ADT-FS will prove to be useful dimensions in measuring the effect of MDT in this context for they are easily corruptible. Nonetheless, we think it important to further establish molecular imaging and SBRT as a first go-to option in oligo-metastatic PCA.

## Conclusion

Early initiation of ADT is still critically debated (61–63) due to the risk of over-treatment and wastage of an effective but also toxic and finite approach. Patients worried about quality-of-life impairments often look for a first treatment step before entering the permanent cycle of systemic therapy. Hence, MDT in oligo-recurrent PCA is a rapidly growing approach (43, 50, 64) aiming to bridge the gap between first-line and systemic treatment. SBRT is swift, locally ablative, and free of relevant AEs. The strengths of this work are the large number of metastases, the uniform treatment regimen, the exclusion of ADT, the very low toxicity rate, and very good ADT-FS in patients with re-SBRTs. Negative prognostic factors could be identified. These patients could be transferred to combination approaches with either stADT or ARPIs.

## Data Availability

The original contributions presented in the study are included in the article/**Supplementary Material**. Further inquiries can be directed to the corresponding author.
